# Poly[[di-μ_3_-nicotinato-hemi-μ_4_-oxalato-hemi-μ_2_-oxalato-neodymium(III)silver(I)] dihydrate]

**DOI:** 10.1107/S1600536809005947

**Published:** 2009-02-25

**Authors:** Xiao-Yan Nie, Qian-Zhu Li

**Affiliations:** a Department of Chemistry, Bijie University, Bijie, Guiyang 551700, People’s Republic of China

## Abstract

The asymmetric unit of the title compound, {[AgNd(C_6_H_4_NO_2_)_2_(C_2_O_4_)]·2H_2_O}_*n*_, contains one Nd^III^ atom, one Ag^I^ atom, one oxalate ligand, two nicotinate ligands and two uncoordinated water mol­ecules. The Nd^III^ atom is eight-coordinated in a distorted square-anti­prismatic coordination geometry by four O atoms from two oxalate ligands and four O atoms from four nicotinate ligands. The Ag^I^ atom has a T-shaped configuration, defined by two N atoms from two nicotinate ligands and one O atom from one oxalate ligand. The nicotinate and oxalate ligands link the Nd and Ag atoms into a three-dimensional coordination framework. O—H⋯O and O—H⋯N hydrogen bonds donated by water mol­ecules are observed in the crystal.

## Related literature

For general background, see: Barbour (2006 ▶); Cheng *et al.* (2007*a* ▶,*b* ▶); Kepert (2006 ▶); Kong *et al.* (2008 ▶); Luo *et al.* (2006 ▶, 2007 ▶); Rao *et al.* (2004 ▶); Zhang *et al.* (2005 ▶). For related structures, see: Arnold *et al.* (1997 ▶); Hartshorn & Steel (1996 ▶); Song & Mao (2005 ▶).
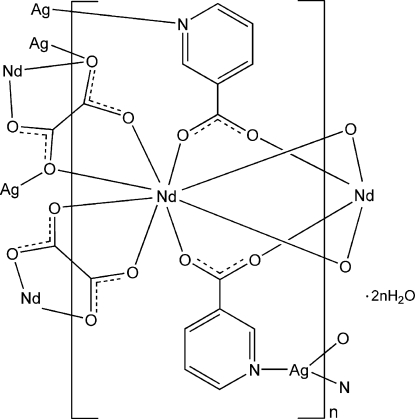


## Experimental

### 

#### Crystal data

[AgNd(C_6_H_4_NO_2_)_2_(C_2_O_4_)]·2H_2_O
*M* *_r_* = 620.37
Monoclinic, 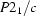

*a* = 9.7441 (1) Å
*b* = 22.4015 (4) Å
*c* = 9.2050 (1) Å
β = 116.992 (1)°
*V* = 1790.42 (4) Å^3^
*Z* = 4
Mo *K*α radiation
μ = 4.02 mm^−1^
*T* = 296 K
0.30 × 0.25 × 0.21 mm

#### Data collection

Bruker APEXII CCD diffractometer
Absorption correction: multi-scan (*SADABS*; Sheldrick, 1996 ▶) *T*_min_ = 0.317, *T*_max_ = 0.439
13299 measured reflections
3202 independent reflections
2645 reflections with *I* > 2σ(*I*)
*R*_int_ = 0.058

#### Refinement

*R*[*F* ^2^ > 2σ(*F* ^2^)] = 0.031
*wR*(*F* ^2^) = 0.072
*S* = 1.05
3202 reflections
253 parameters
6 restraints
H-atom parameters constrained
Δρ_max_ = 1.05 e Å^−3^
Δρ_min_ = −0.74 e Å^−3^

### 

Data collection: *APEX2* (Bruker, 2007 ▶); cell refinement: *SAINT* (Bruker, 2007 ▶); data reduction: *SAINT*; program(s) used to solve structure: *SHELXS97* (Sheldrick, 2008 ▶); program(s) used to refine structure: *SHELXL97* (Sheldrick, 2008 ▶); molecular graphics: *SHELXTL* (Sheldrick, 2008 ▶) and *DIAMOND* (Brandenburg & Putz, 1999 ▶); software used to prepare material for publication: *SHELXTL*.

## Supplementary Material

Crystal structure: contains datablocks I, global. DOI: 10.1107/S1600536809005947/hy2180sup1.cif

Structure factors: contains datablocks I. DOI: 10.1107/S1600536809005947/hy2180Isup2.hkl

Additional supplementary materials:  crystallographic information; 3D view; checkCIF report

## Figures and Tables

**Table 1:** Selected bond lengths (Å)

Ag1—N1^i^	2.176 (5)
Ag1—N2	2.180 (5)
Ag1—O5	2.491 (3)
Nd1—O4^ii^	2.387 (4)
Nd1—O8^i^	2.443 (3)
Nd1—O1	2.449 (4)
Nd1—O3^iii^	2.450 (4)
Nd1—O2^iv^	2.451 (4)
Nd1—O5	2.464 (3)
Nd1—O7	2.491 (3)
Nd1—O6^v^	2.525 (3)

Symmetry codes: (i) 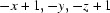; (ii) 
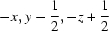
; (iii) 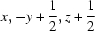; (iv) 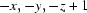; (v) 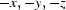.

**Table 2:** Hydrogen-bond geometry (Å, °)

*D*—H⋯*A*	*D*—H	H⋯*A*	*D*⋯*A*	*D*—H⋯*A*
O2*W*—H3*W*⋯N1^vi^	0.84	2.63	3.308 (9)	138
O2*W*—H4*W*⋯O1*W*^vii^	0.84	1.99	2.794 (10)	160
O1*W*—H2*W*⋯O7	0.84	2.12	2.962 (7)	176
O1*W*—H1*W*⋯O2*W*^viii^	0.84	2.07	2.890 (9)	164

Symmetry codes: (vi) 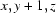; (vii) 
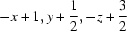
; (viii) 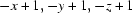.

**Fig. 1.**
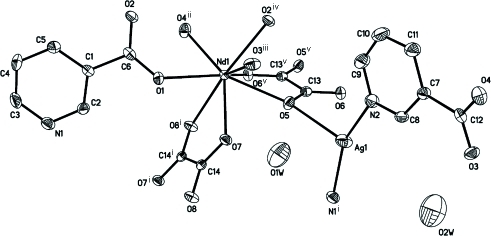
The asymmetric unit of the title compound, together with symmetry-related atoms to complete the coordination units. Displacement ellipsoids are drawn at the 50% probability level. [Symmetry codes: (i) -x+1, -y, -z+1; (ii) -x, y-1/2, -z+1/2; (iii) x, -y+1/2, z+1/2; (iv) -x, -y, -z+1; (v) -x, -y, -z.]

**Fig. 2.**
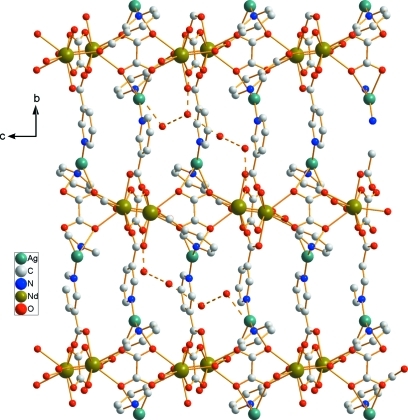
View of the structure along the a axis. Dashed lines denote hydrogen bonds.

## supplementary crystallographic information

### Comment

The design and construction of transition–lanthanide metal complexes have gained great recognition over the last decade because of their intriguing network topologies and potential applications, and because of their magnetic properties, capacity for gas storage, luminescent properties and so on (Barbour, 2006; Kepert, 2006; Kong *et al.*, 2008; Rao *et al.*, 2004; Zhang *et al.*, 2005). Nicotinic acid is a multifunctional bridging ligand possessing of O and N donors, which can thus be utilized to construct transition–lanthanide heterometallic complexes *via* the carboxyl O atoms binding to lanthanides and N atoms bonding to transition metal ions such as Ag^I^ or Cu^I^ (Cheng *et al.*, 2007a,b; Luo *et al.*, 2006, 2007). On the basis of above considerations, a new three-dimensional 4d-4f coordination framework was obtained from the hydrothermal treatment of Nd_2_O_3_, AgNO_3_, oxalic acid, nicotinic acid and nitric acid in water.

As depicted in Fig. 1, the title compound contains one Nd^III^ atom, one Ag^I^ atom, one oxalate (1/2 + 1/2) ligand, two nicotinate ligands and two uncoordinated water molecules in the asymmetric unit. The Nd^III^ atom is eight-coordinated in a distorted square-antiprismatic coordination geometry by four O atoms from two oxalate ligands and four O atoms from four nicotinate ligands (Table 1), with Nd—O bond lengths and O—Nd—O bond angles ranging from 2.387 (4) to 2.525 (3) Å and 64.96 (11) to 148.77 (12)°, respectively, all of which are within the range of those observed for other eight-coordinated Nd^III^ complexes with O donor ligands (Song & Mao, 2005). The Ag^I^ atom is located in a T-shaped configuration, defined by two N atoms from two nicotinate lignads and one O atom from one oxalate lignad. The Ag—N and Ag—O bond distances and the N—Ag—N angle are similar to those of other Ag^I^ structures with T-shaped configuration (Hartshorn & Steel, 1996; Arnold *et al.*, 1997).

In the crystal structure, the Nd and Ag atoms are linked by the carboxylate groups of nicotinate ligands and the oxalate ligands, forming a two-dimensional layer in the *ac* plane. The shortest Nd···Nd distance is 4.224 (3) Å. The layers are further constructed into a three-dimensional coordination framework through bridging nicotinate ligands (Fig. 2). O—H···O and O—H···N hydrogen bonds donated by water molecules are observed (Table 2).

### Experimental

A mixture of Nd_2_O_3_ (0.168 g, 0.5 mmol), AgNO_3_ (0.169 g, 1 mmol), nicotinic acid (0.123 g, 1 mmol), oxalic acid (0.090 g, 1 mmol), HNO_3_ (0.12 ml) and H_2_O (10 ml) was placed in a 23 ml Teflon-lined reactor, which was heated to 433 K for 3 d and then cooled to room temperature at a rate of 10 K h^-1^. The pale-purple plate crystals obtained were washed with water and dried in air (yield 46% based on Nd).

### Refinement

Carbon-bound H atoms were positioned geometrically and treated as riding on the parent C atoms with C—H = 0.93 Å, and with *U*_iso_(H) = 1.2*U*_eq_(C). Water H atoms were tentatively located in difference Fourier maps and were fixed with distance of O—H = 0.84 Å and *U*_iso_(H) = 1.5*U*_eq_(O). The hightest peak is located 0.92 Å from Nd1 and the deepest hole is located 0.81 Å from Ag1.

### Crystal data

**Table:**

[AgNd(C_6_H_4_NO_2_)_2_(C_2_O_4_)]·2H_2_O	*F*(000) = 1188
*M**_r_* = 620.37	*D*_x_ = 2.301 Mg m^−^^3^
Monoclinic, *P*2_1_/*c*	Mo *K*α radiation, λ = 0.71073 Å
Hall symbol: -P 2ybc	Cell parameters from 5837 reflections
*a* = 9.7441 (1) Å	θ = 2.8–27.9°
*b* = 22.4015 (4) Å	µ = 4.02 mm^−^^1^
*c* = 9.2050 (1) Å	*T* = 296 K
β = 116.992 (1)°	Block, pale-purple
*V* = 1790.42 (4) Å^3^	0.30 × 0.25 × 0.21 mm
*Z* = 4	

### Data collection

**Table:**

Bruker APEXII CCD diffractometer	3202 independent reflections
Radiation source: fine-focus sealed tube	2645 reflections with *I* > 2σ(*I*)
graphite	*R*_int_ = 0.058
φ and ω scans	θ_max_ = 25.2°, θ_min_ = 2.4°
Absorption correction: multi-scan (*SADABS*; Sheldrick, 1996)	*h* = −11→11
*T*_min_ = 0.317, *T*_max_ = 0.439	*k* = −26→26
13299 measured reflections	*l* = −11→11

### Refinement

**Table:**

Refinement on *F*^2^	Primary atom site location: structure-invariant direct methods
Least-squares matrix: full	Secondary atom site location: difference Fourier map
*R*[*F*^2^ > 2σ(*F*^2^)] = 0.031	Hydrogen site location: inferred from neighbouring sites
*wR*(*F*^2^) = 0.072	H-atom parameters constrained
*S* = 1.05	*w* = 1/[σ^2^(*F*_o_^2^) + (0.0246*P*)^2^ + 1.8836*P*] where *P* = (*F*_o_^2^ + 2*F*_c_^2^)/3
3202 reflections	(Δ/σ)_max_ < 0.001
253 parameters	Δρ_max_ = 1.05 e Å^−^^3^
6 restraints	Δρ_min_ = −0.74 e Å^−^^3^

### Fractional atomic coordinates and isotropic or equivalent isotropic displacement parameters (Å^2^)

**Table:**

	*x*	*y*	*z*	*U*_iso_*/*U*_eq_	
Ag1	0.17888 (6)	0.14737 (2)	0.01878 (7)	0.05145 (17)	
C1	0.3858 (6)	−0.0646 (2)	0.9171 (6)	0.0253 (11)	
N1	0.6264 (5)	−0.1161 (2)	1.0156 (6)	0.0364 (11)	
Nd1	0.14173 (3)	0.008872 (12)	0.38289 (3)	0.01977 (10)	
O1	0.2914 (4)	−0.00473 (16)	0.6779 (4)	0.0309 (9)	
O1W	0.3918 (8)	0.1959 (3)	0.4391 (7)	0.098 (2)	
H1W	0.4095	0.2081	0.3629	0.147*	
H2W	0.3907	0.1585	0.4384	0.147*	
C2	0.5159 (6)	−0.0809 (2)	0.9055 (7)	0.0320 (13)	
H2	0.5286	−0.0671	0.8172	0.038*	
N2	0.0364 (5)	0.2168 (2)	0.0474 (6)	0.0367 (11)	
O2	0.1337 (4)	−0.02728 (19)	0.7871 (4)	0.0361 (9)	
O2W	0.4855 (9)	0.7657 (3)	0.7785 (9)	0.119 (3)	
H4W	0.5387	0.7427	0.8560	0.178*	
H3W	0.4683	0.7974	0.8159	0.178*	
C3	0.6065 (7)	−0.1347 (3)	1.1434 (7)	0.0426 (15)	
H3	0.6804	−0.1597	1.2194	0.051*	
O3	0.1134 (4)	0.38638 (16)	−0.0591 (5)	0.0368 (10)	
C4	0.4824 (7)	−0.1187 (3)	1.1673 (7)	0.0402 (15)	
H4	0.4745	−0.1310	1.2597	0.048*	
O4	−0.0282 (5)	0.42752 (17)	0.0429 (5)	0.0371 (9)	
C5	0.3706 (7)	−0.0841 (3)	1.0515 (7)	0.0339 (13)	
H5	0.2838	−0.0736	1.0633	0.041*	
O5	0.1169 (4)	0.05546 (16)	0.1296 (4)	0.0315 (9)	
C6	0.2600 (6)	−0.0288 (2)	0.7832 (6)	0.0278 (12)	
O6	−0.0401 (4)	0.05896 (15)	−0.1370 (4)	0.0278 (8)	
C7	−0.0146 (6)	0.3216 (2)	0.0401 (6)	0.0276 (12)	
O7	0.3853 (4)	0.06386 (16)	0.4497 (4)	0.0278 (8)	
C8	0.0645 (6)	0.2733 (2)	0.0224 (7)	0.0305 (13)	
H8	0.1414	0.2804	−0.0087	0.037*	
O8	0.6416 (4)	0.05526 (16)	0.5788 (4)	0.0298 (9)	
C9	−0.0735 (7)	0.2077 (3)	0.0938 (8)	0.0465 (16)	
H9	−0.0935	0.1687	0.1131	0.056*	
C10	−0.1580 (8)	0.2526 (3)	0.1142 (9)	0.0530 (18)	
H10	−0.2342	0.2441	0.1455	0.064*	
C11	−0.1288 (7)	0.3105 (3)	0.0878 (7)	0.0409 (15)	
H11	−0.1844	0.3419	0.1017	0.049*	
C12	0.0257 (6)	0.3829 (2)	0.0058 (6)	0.0265 (12)	
C13	0.0207 (6)	0.0329 (2)	−0.0029 (6)	0.0246 (11)	
C14	0.5076 (6)	0.0349 (2)	0.5090 (6)	0.0224 (11)	

### Atomic displacement parameters (Å^2^)

**Table:**

	*U*^11^	*U*^22^	*U*^33^	*U*^12^	*U*^13^	*U*^23^
Ag1	0.0384 (3)	0.0263 (3)	0.0940 (4)	0.0076 (2)	0.0339 (3)	0.0094 (2)
C1	0.021 (3)	0.024 (3)	0.028 (3)	0.002 (2)	0.008 (2)	−0.002 (2)
N1	0.028 (3)	0.034 (3)	0.046 (3)	0.005 (2)	0.016 (2)	0.006 (2)
Nd1	0.01772 (16)	0.02063 (17)	0.02011 (15)	0.00101 (11)	0.00786 (12)	0.00165 (11)
O1	0.028 (2)	0.038 (2)	0.0226 (19)	0.0006 (17)	0.0074 (17)	0.0061 (16)
O1W	0.122 (6)	0.076 (4)	0.107 (5)	−0.015 (4)	0.060 (5)	0.011 (4)
C2	0.032 (3)	0.030 (3)	0.039 (3)	0.006 (3)	0.020 (3)	0.010 (3)
N2	0.033 (3)	0.023 (3)	0.053 (3)	0.000 (2)	0.019 (3)	0.004 (2)
O2	0.021 (2)	0.056 (3)	0.029 (2)	0.0094 (19)	0.0094 (18)	0.0049 (18)
O2W	0.162 (7)	0.076 (5)	0.122 (6)	−0.005 (5)	0.067 (6)	−0.004 (4)
C3	0.037 (4)	0.041 (4)	0.038 (4)	0.011 (3)	0.007 (3)	0.015 (3)
O3	0.038 (2)	0.025 (2)	0.059 (3)	−0.0018 (18)	0.031 (2)	0.0047 (18)
C4	0.054 (4)	0.041 (4)	0.025 (3)	0.007 (3)	0.017 (3)	0.009 (3)
O4	0.045 (2)	0.026 (2)	0.037 (2)	0.0057 (19)	0.015 (2)	−0.0050 (17)
C5	0.031 (3)	0.040 (4)	0.034 (3)	0.003 (3)	0.018 (3)	0.000 (3)
O5	0.035 (2)	0.033 (2)	0.0225 (19)	−0.0102 (18)	0.0096 (18)	−0.0002 (16)
C6	0.022 (3)	0.027 (3)	0.028 (3)	0.003 (2)	0.005 (3)	−0.005 (2)
O6	0.031 (2)	0.024 (2)	0.0236 (19)	0.0007 (16)	0.0081 (17)	0.0009 (15)
C7	0.028 (3)	0.022 (3)	0.036 (3)	−0.002 (2)	0.017 (3)	−0.001 (2)
O7	0.022 (2)	0.025 (2)	0.036 (2)	0.0043 (16)	0.0123 (18)	0.0038 (16)
C8	0.025 (3)	0.026 (3)	0.045 (3)	0.001 (2)	0.019 (3)	0.003 (2)
O8	0.017 (2)	0.030 (2)	0.037 (2)	−0.0010 (16)	0.0078 (18)	−0.0072 (16)
C9	0.047 (4)	0.030 (4)	0.068 (5)	−0.009 (3)	0.031 (4)	0.009 (3)
C10	0.052 (4)	0.048 (4)	0.077 (5)	−0.001 (3)	0.046 (4)	0.011 (4)
C11	0.039 (4)	0.037 (4)	0.054 (4)	0.004 (3)	0.028 (3)	0.003 (3)
C12	0.024 (3)	0.024 (3)	0.025 (3)	0.006 (2)	0.006 (3)	0.000 (2)
C13	0.025 (3)	0.026 (3)	0.027 (3)	0.001 (2)	0.015 (3)	−0.002 (2)
C14	0.024 (3)	0.032 (3)	0.016 (2)	0.003 (2)	0.012 (2)	0.001 (2)

### Geometric parameters (Å, °)

**Table:**

Ag1—N1^i^	2.176 (5)	O2W—H3W	0.8400
Ag1—N2	2.180 (5)	C3—C4	1.371 (8)
Ag1—O5	2.491 (3)	C3—H3	0.9300
C1—C2	1.369 (7)	O3—C12	1.247 (6)
C1—C5	1.382 (7)	C4—C5	1.368 (8)
C1—C6	1.514 (7)	C4—H4	0.9300
N1—C3	1.343 (7)	O4—C12	1.247 (6)
N1—C2	1.348 (7)	C5—H5	0.9300
Nd1—O4^ii^	2.387 (4)	O5—C13	1.259 (6)
Nd1—O8^i^	2.443 (3)	O6—C13	1.246 (6)
Nd1—O1	2.449 (4)	C7—C8	1.382 (7)
Nd1—O3^iii^	2.450 (4)	C7—C11	1.391 (7)
Nd1—O2^iv^	2.451 (4)	C7—C12	1.501 (7)
Nd1—O5	2.464 (3)	O7—C14	1.244 (6)
Nd1—O7	2.491 (3)	C8—H8	0.9300
Nd1—O6^v^	2.525 (3)	O8—C14	1.251 (6)
O1—C6	1.262 (6)	C9—C10	1.366 (9)
O1W—H1W	0.8400	C9—H9	0.9300
O1W—H2W	0.8400	C10—C11	1.374 (9)
C2—H2	0.9300	C10—H10	0.9300
N2—C9	1.336 (7)	C11—H11	0.9300
N2—C8	1.336 (7)	C13—C13^v^	1.536 (11)
O2—C6	1.248 (6)	C14—C14^i^	1.572 (11)
O2W—H4W	0.8400		
			
N1^i^—Ag1—N2	153.22 (18)	H4W—O2W—H3W	109.4
N1^i^—Ag1—O5	100.53 (15)	N1—C3—C4	123.2 (5)
N2—Ag1—O5	104.74 (15)	N1—C3—H3	118.4
C2—C1—C5	117.7 (5)	C4—C3—H3	118.4
C2—C1—C6	120.9 (5)	C12—O3—Nd1^vi^	109.5 (3)
C5—C1—C6	121.3 (5)	C5—C4—C3	118.2 (5)
C3—N1—C2	117.2 (5)	C5—C4—H4	120.9
C3—N1—Ag1^i^	120.6 (4)	C3—C4—H4	120.9
C2—N1—Ag1^i^	122.1 (4)	C12—O4—Nd1^vii^	176.5 (4)
O4^ii^—Nd1—O8^i^	89.54 (13)	C4—C5—C1	120.3 (5)
O4^ii^—Nd1—O1	73.01 (13)	C4—C5—H5	119.8
O8^i^—Nd1—O1	74.10 (12)	C1—C5—H5	119.8
O4^ii^—Nd1—O3^iii^	123.80 (13)	C13—O5—Nd1	117.2 (3)
O8^i^—Nd1—O3^iii^	135.02 (12)	C13—O5—Ag1	98.5 (3)
O1—Nd1—O3^iii^	86.96 (13)	Nd1—O5—Ag1	143.11 (15)
O4^ii^—Nd1—O2^iv^	78.22 (13)	O2—C6—O1	126.6 (5)
O8^i^—Nd1—O2^iv^	144.59 (13)	O2—C6—C1	115.8 (5)
O1—Nd1—O2^iv^	131.08 (12)	O1—C6—C1	117.5 (5)
O3^iii^—Nd1—O2^iv^	77.22 (14)	C13—O6—Nd1^v^	115.0 (3)
O4^ii^—Nd1—O5	137.30 (12)	C8—C7—C11	117.8 (5)
O8^i^—Nd1—O5	95.33 (12)	C8—C7—C12	118.6 (5)
O1—Nd1—O5	148.77 (12)	C11—C7—C12	123.6 (5)
O3^iii^—Nd1—O5	80.10 (13)	C14—O7—Nd1	117.7 (3)
O2^iv^—Nd1—O5	73.50 (12)	N2—C8—C7	123.6 (5)
O4^ii^—Nd1—O7	145.13 (12)	N2—C8—H8	118.2
O8^i^—Nd1—O7	65.86 (12)	C7—C8—H8	118.2
O1—Nd1—O7	76.51 (12)	C14—O8—Nd1^i^	119.5 (3)
O3^iii^—Nd1—O7	70.23 (12)	N2—C9—C10	123.5 (6)
O2^iv^—Nd1—O7	135.96 (12)	N2—C9—H9	118.3
O5—Nd1—O7	72.38 (12)	C10—C9—H9	118.3
O4^ii^—Nd1—O6^v^	75.69 (12)	C9—C10—C11	119.0 (6)
O8^i^—Nd1—O6^v^	74.76 (11)	C9—C10—H10	120.5
O1—Nd1—O6^v^	135.45 (12)	C11—C10—H10	120.5
O3^iii^—Nd1—O6^v^	137.26 (12)	C10—C11—C7	119.0 (6)
O2^iv^—Nd1—O6^v^	70.06 (12)	C10—C11—H11	120.5
O5—Nd1—O6^v^	64.96 (11)	C7—C11—H11	120.5
O7—Nd1—O6^v^	117.43 (11)	O4—C12—O3	123.2 (5)
C6—O1—Nd1	132.4 (3)	O4—C12—C7	119.5 (5)
H1W—O1W—H2W	109.0	O3—C12—C7	117.4 (5)
N1—C2—C1	123.4 (5)	O6—C13—O5	125.6 (5)
N1—C2—H2	118.3	O6—C13—C13^v^	118.0 (6)
C1—C2—H2	118.3	O5—C13—C13^v^	116.3 (6)
C9—N2—C8	117.2 (5)	O7—C14—O8	127.1 (5)
C9—N2—Ag1	125.2 (4)	O7—C14—C14^i^	116.6 (5)
C8—N2—Ag1	117.6 (4)	O8—C14—C14^i^	116.3 (5)
C6—O2—Nd1^iv^	142.6 (3)		

Symmetry codes: (i) −*x*+1, −*y*, −*z*+1; (ii) −*x*, *y*−1/2, −*z*+1/2; (iii) *x*, −*y*+1/2, *z*+1/2; (iv) −*x*, −*y*, −*z*+1; (v) −*x*, −*y*, −*z*; (vi) *x*, −*y*+1/2, *z*−1/2; (vii) −*x*, *y*+1/2, −*z*+1/2.

### Hydrogen-bond geometry (Å, °)

**Table:**

*D*—H···*A*	*D*—H	H···*A*	*D*···*A*	*D*—H···*A*
O2W—H3W···N1^viii^	0.84	2.63	3.308 (9)	138
O2W—H4W···O1W^ix^	0.84	1.99	2.794 (10)	160
O1W—H2W···O7	0.84	2.12	2.962 (7)	176
O1W—H1W···O2W^x^	0.84	2.07	2.890 (9)	164

Symmetry codes: (viii) *x*, *y*+1, *z*; (ix) −*x*+1, *y*+1/2, −*z*+3/2; (x) −*x*+1, −*y*+1, −*z*+1.
